# Seeing the unseen: Eiichi Nakamura on chemistry, life, and a new chapter in China

**DOI:** 10.1093/nsr/nwag400

**Published:** 2026-07-01

**Authors:** Weijie Zhao

## Abstract

Walking into the meeting room for the interview with *National Science Review* (NSR), Prof. Eiichi Nakamura (中村栄一) first played us a documentary about himself that ran over 20 minutes. The footage was, of course, related to chemistry, but it was far more about art and life: his passion for the Baroque flute, his small concerts with his wife and friends, his travel footprints spanning the globe since the age of 20, and his friendships with numerous fellow scientists.

Over the 2-hour interview that followed, the 75-year-old professor left a profound impression on us—romantic yet rigorous, restrained yet humorous.

Prof. Nakamura is a world-renowned organic chemist and molecular imaging chemist. Using high-resolution transmission electron microscopy (TEM) to visualize real-time chemical reactions, he has pushed open the door to ‘seeing is believing’ at the single-molecule level in organic chemistry research. He proposed the ‘Element Strategy’, which received support from the Japanese and other governments, aiming to make the industrial chemistry greener, cleaner, and more sustainable.

After 30 years at the University of Tokyo, Prof. Nakamura left Japan this March and joined full-time the Nankai University in Tianjin, China, where he is now embarking on brand new research topics. In this interview, you will read about Prof. Nakamura’s experiences, achievements, and visions in chemical research—and, perhaps more importantly, you will discover a life philosophy of ‘living in the present’ and ‘make the best of what comes’ that permeates his rich life story.

## Teaser

Prof. Nakamura's experiences, achievements, and visions in chemical researchand, perhaps more importantly, his life philosophy.

## SEEING IS BELIEVING: MAKE CHEMICAL REACTIONS VISIBLE

***NSR*:** How did you become a chemist? What were your original research interests and how did you come to use electron microscopy in your research?

***Nakamura*:** That is a long story. Life is extremely stochastic, so I always say things are ‘beyond expectation’. When I entered college, my choices were architecture or chemistry, because I was very interested in building things. During my primary school period, I hand-built wooden 1/1000-scale model battleships of the Japanese navy; and during my high school and college years, I was extremely interested in railways and built 1/80 brass models of trains and steam locomotives. I even published an academic paper on the history of Israel railway when I was 21.

I did well in high school and was confident that I would be admitted to the University of Tokyo. However, in the year 1969, the Zenkyoto Movement was going on—students occupied campuses and the University of Tokyo entrance exam was cancelled. So, I took the exam of the Tokyo Institute of Technology (TIT) and went there.

Prof. Teruaki Mukaiyama (向山光昭), a synthetic organic professor in his early forties, was the best chemistry professor at TIT. I chose him to be my mentor, because synthesis, which means making molecules, well aligned with my own interests.

**Figure 1:**
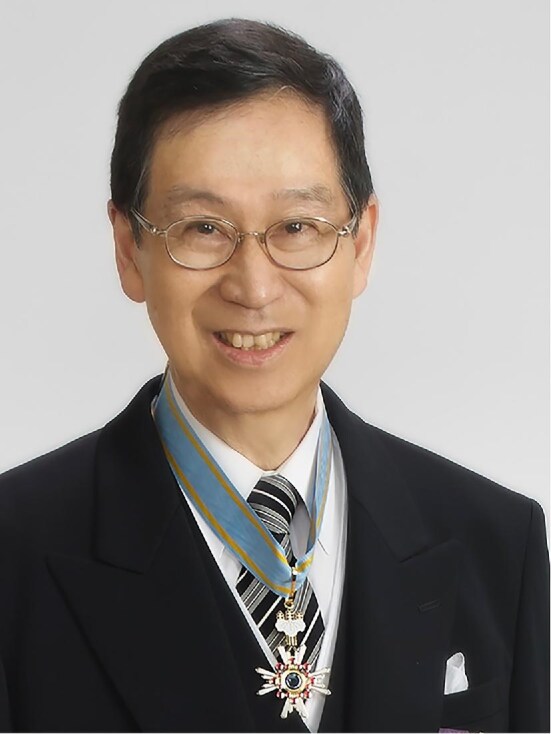
Professor Eiichi Nakamura with the Order of the Sacred Treasure (Gold Rays with Neck Ribbon), 2024. Photo provided by Prof. Nakamura.

Prof. Mukaiyama was famous as a synthetic organic chemist, but actually he was trained as a physical organic chemist and had an interest in the mechanism of reactions. This influenced me and I also became interested in mechanisms. At that time, reaction mechanisms are invisible—you cannot see the molecules, but only guess at the mechanisms. For a long time, like all chemists, I hoped to see molecules.

In 1989, when I was 38, I asked Prof. Keiji Morokuma (諸熊奎治), an eminent quantum theoretical chemist, to do some simulation for my project, and he suggested: ‘Why don’t you do it yourself?’ So, when I was 39, I started to do computational chemistry with the then mainframe computers, which are very large but slower than today’s iPhones.

Gradually, I became unsatisfied with virtual simulations. As an experimental chemist, I didn’t like virtual images and wanted to see real molecules and their reactions. Then what beyond expectation happened in 1998. I shared a hotel room for two nights with Prof. Sumio Iijima (飯島澄男), who discovered carbon nanotubes and is a world-renowned electron microscopist. I told him ‘I have a dream of observing molecules with an electron microscope’, and he said: ‘It is impossible. Organic molecules decompose under electron beams.’

But we collaborated. During 3 years of collaboration, I was convinced he was wrong—organic molecules do not decompose so easily. Strong beams burn samples, but weak beams for microscopy don’t decompose organic molecules so readily. We published a *Science* paper in 2007, demonstrating we could see single organic molecules in motion [Koshina M *et al. Science* 2007; **316**: 853].

So that is the story, and to make it short: I started with synthetic organic chemistry to make compounds, then partly because of Prof. Mukaiyama I became interested in reaction mechanisms, then to theoretical simulation which is virtual, and then eventually I turned to electron microscopy, believing we could see molecules. It took 20 years from the beginning. I evolved gradually—and if I hadn’t shared a room with Iijima, I never would have started electron microscopy.

*I told him ‘I have a dream of observing molecules with an electron microscope’, and he said: ‘It is impossible.’*

*—Eiichi Nakamura*

***NSR*:** Is it technically difficult to see the organic molecules through TEM?

***Nakamura*:** It is very difficult. Single-molecule imaging needs special touch and sensibility. To this day, only a few groups in the world can routinely do it.

Single molecules move rapidly in solution, and we need to chemically attach them onto a carrier so that they are fixed

**Figure 2:**
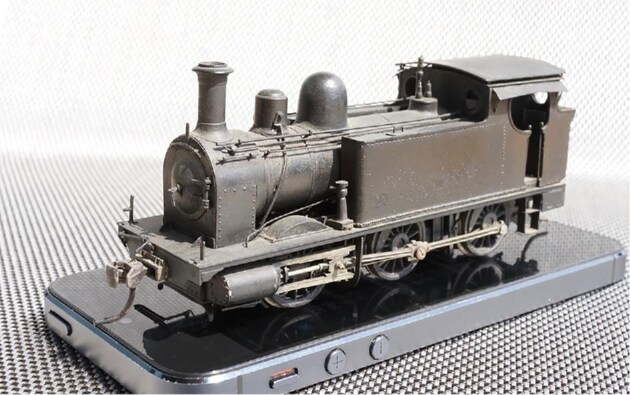
A 1/80 brass railway model on iPhone 5, hand-made by Prof. Nakamura in his high school years. Photo provided by Prof. Nakamura.

loosely and their motions can be observed in vacuum environment under the microscope. Our success depends on our chemical technique to take organic molecules out of solution and onto the carrier. This is essentially single-molecule synthetic chemistry.

***NSR*:** What are your group’s major contributions in the field?

***Nakamura*:** The first important achievement is the abovementioned 2007 *Science* paper, entitled ‘Imaging of Single Organic Molecules in Motion’. This paper was rarely cited for almost 10 years because it violated electron microscopists’ conventional expectation that organic molecules would decompose under an electron beam.

The second important paper was published in the *Journal of the American Chemical Society* (*JACS*) in 2017, entitled ‘Direct Microscopic Analysis of Individual C_60_ Dimerization Events: Kinetics and Mechanisms’ [Okada S *et al. JACS* 2017; **139**: 18281–7]. We saw 20 molecules reacted individually within 200 seconds, one after another. Their lifetimes are totally different: the shortest is like a baby passing away at 6 months old, while the longest is like an old man living to 100 years old. We accurately measured the process and obtained the distribution of the reaction rate—the beginning of our approach to ‘statistical mechanical chemistry’. By statistical mechanical chemistry, I mean studying chemical reactions by analyzing the statistics of many individual molecular events rather than only their ensemble averages.

This is the first time that human can study chemical reaction in a quantitative manner at single-molecule level. I sometimes tell people that what we are doing is like molecular opinion poll. We interview every single molecule one by one, and then reconstruct the overall group behavior. I think chemists have long been too accustomed to state-to-state chemistry, which focuses on the mass behavior but ignores individual molecules, but we should respect every molecular and every reaction pathway, just as we respect individual human choices.

The third breakthrough came two years ago in a 2024 *Science* paper [Liu D *et al. Science* 2024; **384**: 1212–9]. In this work, we demonstrated experimentally that the two definitions of entropy, the Clausius one (*S* = *Q*/*T*) and the Boltzmann one (*S* = *k*_B_ln*Ω*), are equivalent. This equivalence has been theoretically proved long ago, but its experimental verification is extremely difficult. As entropy is a physics concept, this is almost a pure physics research and is fundamentally different from the real-time imaging of single-molecule dynamics.

We performed it by irradiating a crystal with an electron beam. When the crystal is ‘shaken’ by the beam, its structure is disturbed. We measured the disappearance of the crystal’s diffraction peaks caused by the irradiation, and then, by analyzing the speed of the state changes, we estimate the entropy. It is a little bit difficult to explain, but this is the core essence.

*What we are doing is like molecular opinion poll. We interview every single molecule one by one, and then reconstruct the overall group behavior.*

*—Eiichi Nakamura*

***NSR*:** How did you choose the research topic of entropy?

***Nakamura*:** It may seem ‘beyond expectation’ but actually just happened naturally. It was known that under electron beam irradiation, crystal diffraction peaks decay and disappear. People always thought it is decomposition. But since I knew that organic molecules do not decompose under electron beam, it was natural to suppose that crystals, which are made up of multiple molecules, should not decompose either. With this intuition, I did the experiment and proved that I am correct: the crystal does not decompose, but only becomes structurally disordered—it becomes messy instead of being burned into ashes. And more careful measurements and analysis gave raise to the *Science* paper.

***NSR*:** What are your expectations of this field?

***Nakamura*:** I hope that chemists, especially organic chemists, can use electron microscopy as a standard analytical tool. I have witnessed the emergence of successive generations of organic analytical tools. Infrared spectroscopy came first, even before me; then was nuclear magnetic resonance, which came into use in my undergraduate days; the third was X-ray crystallography became a routine tool for organic chemists in the 1980s; the fourth was quantum chemical theoretical calculations came in the 1990s. I believe electron microscopy should become the next standard tool in chemistry. The next tool must be generative artificial intelligence (AI).

I think one major challenge everyone must consider is that we are crossing the border of flasks and test tubes and coming into the field of nanoscale chemistry. Now we use carbon nanotubes as test tubes under electron microscopic conditions, and that is far beyond the ordinary sense of chemists.

## IRON CATALYSIS AND THE ELEMENT STRATEGY

***NSR*:** Besides cinematic chemistry, what are the other significant achievements you have made in chemistry?

***Nakamura*:** That’s of course iron catalysis. Scientifically, TEM in chemistry is my most important contribution; while practically, I believe my most influential contribution is that I made iron catalysis popular and proposed the Element Strategy.

The use of iron (Fe) for catalysis was first pioneered by Morris S. Kharasch in the 1940s in Chicago, but then this line of research was stopped. Then Jay Kochi, a Japanese–American chemist, also studied iron catalysis, but he also stopped his research in the 1970s. After that, there were few efforts on iron catalysis until in 2000, we published an important paper entitled ‘Iron-Catalyzed Olefin Carbometalation’ [Nakamura M *et al. JACS* 2000; **122**: 978–9]. Then, we published another influential paper in 2004 [Nakamura M *et al. JACS* 2004; **126**: 3686–7], and then a third one in 2008 on C-H activation [Norinder J *et al. JACS* 2008; **130**: 5858–9], proving that new types of chemical reactions can be realized using iron catalysis. Our series of papers gradually attracted the academic community’s attention and attracted more researchers into this field.

Iron is the most abundant heavy metal in the universe, because it is the end product of stellar nucleosynthesis. As all living organisms have evolved and survived in iron-rich environment, iron is also non-toxic to life.

Iron is abundant, non-toxic and sustainable for long-term use. However, iron is difficult to control chemically because it is a 3*d* transition metal. Among 3*d*, 4*d*, and 5*d* orbitals, iron has an extremely small ligand-field splitting energy. Its electronic spin state can easily switch back and forth spontaneously, which makes it extremely difficult to manipulate and apply in catalysis. I don’t think anyone has yet found a perfect way to fully utilize iron’s catalytic potential.

Based on my studies on iron catalysis, I also proposed the Element Strategy concept in 2004 and submitted it to the Japanese government. Later, the United States and China also adopted similar initiatives.

The core idea is to reduce the consumption of precious metals, replace precious metals with abundant elements such as iron, and promote resource recycling. This idea is very appealing to policy makers, even if most organic chemists did not immediately understand it. But gradually everyone realized that chemical elements are non-renewable and need rational utilization.

***NSR*:** How did you become interested in the ‘cheap metals’?

***Nakamura*:** This has long been my research interest. Before I started this direction, there was a senior colleague in the lab next to mine, Prof. Jiro Tsuji (辻二郎), a founder of palladium catalysis, who was fully worthy of a Nobel Prize but never received it. I deliberately decided not to compete in palladium and precious metal research—as a student, you cannot compete with those top pioneers. Therefore, throughout my career, I focused on copper, zinc, nickel and iron, the cheap metals for research. These research directions cost less funding, and I always choose fields that others overlook instead of following mainstream trends. I go to somewhere that people are not interested in.

*The core idea is to reduce the consumption of precious metals, replace precious metals with abundant elements such as iron, and promote resource recycling.*

*—Eiichi Nakamura*

## INDUSTRIAL TRANSLATION: FAILED AND SUCCESSFUL CASES

***NSR*:** You have said that scientists should provide solutions to social problems. Do you have some examples that your findings served the chemical industry and the society?

***Nakamura*:** It is not easy to list specific cases clearly, but after our initial publications in the early 2000s, many companies began to adopt iron catalysis technology, including Merck in New Jersey. Everyone now recognizes that iron can be a very useful catalyst.

We also attempted to translate our research into industrial production, though most attempts ended in failure, and that is perfectly acceptable.

One example is that we worked on organic solar cells from 2004 to 2015. We started early in this field and greatly aroused the research interest across Japan. We collaborated with a chemical company to develop printable plastic solar cells and produced around 1000 trial samples, yet the energy efficiency was still insufficient and both we and the company decided to stop the project. Even now, perovskite solar cell products have not entered the market profitably; it was indeed very challenging research.

We also developed some pharmaceutical candidate compounds; one candidate even went into animal testing, but it failed due to toxicity in dog trials. We have also collaborated with generic pharmaceutical companies on drug formulation research, and one related product is likely to be launched on the market soon.

I have filed more than 100 patents in total from my academic work. Only one of them ever brought me real economic return, just enough to buy a car. The purpose of patenting is not mainly to make money, but to protect intellectual property and give companies a sense of security for long-term cooperation. I maintain cooperation with many companies, but I do not spend too much personal time on industrial projects. I let my students and others take charge instead.

Apart from academic research itself, by using TEM, we have produced a series of educational chemistry videos for high schools. These materials have already been used in Japan and I hope the Chinese government can also adopt our videos for chemistry education. We would be glad to produce a Chinese version. The core purpose is to let young generations know chemistry can be visible and this can completely change their understanding of chemistry.

## GENERATIVE AI: A NEW FRIEND OF CHEMISTS

***NSR*:** We know you are very interested in AI. In your view, will AI replace most of chemists’ work, or will it merely serve as a tool?

***Nakamura*:** It depends on how good is the chemist. Good chemists with solid intelligence and great experimental skills will never be replaced by AI, and I believe generative AI will be a good friend of them.

ChatGPT launched at the end of November 2022, and I started using it immediately. In the second week of December, I told the undergraduate students in the organic chemistry class that they must try ChatGPT, for it is extremely helpful for chemistry study. I gave them a practical example: I asked ChatGPT to summarize the latest research progress on Grignard addition to ketones within 200 words, either in Japanese or English, and it gave an almost perfect answer in just 10 seconds—though not entirely error-free in every detail. I told my class: this guy is much better than you; you need at least 1 week to do it and now the answer comes in less than 10 seconds.

I even adopted AI as a teaching tool. Barry Sharpless won his second Nobel Prize in 2022. I know Sharpless quite well, so I assigned undergraduates an essay question: analyze the reasons why Sharpless could win two Nobel Prizes, and what kind of research mindsets are needed to achieve such accomplishments. This is an extremely difficult topic for second-year undergraduates, even challenging for senior researchers. With the help of AI, 60 students took the assignment and several of them produced excellent analyses and discussions about academic creativity and research orientation. It turned out to be a very valuable educational exercise for top students.

***NSR*:** In which way can generative AI help on chemical research?

***Nakamura*:** I think AI may assist us to choose research topics. We may brainstorm with AI to figure out the valuable problems that need be solved, and can be solved in maybe 10 years. Ideally, we should discover topics that seem impossible now but can be broken through within 1 or 2 years.

AI is good at sorting out existing literature and tell you what have been done, but it cannot directly tell you what is still missing—that is the same as humans; so you have to frame your questions very precisely. This is very challenging, and it is a direction worth exploring through human–AI collaboration. Human researchers can definitely train and optimize AI models for chemistry research.

***NSR*:** Can AI technically improve the electron microscopy technology?

***Nakamura*:** Electron microscopy relies heavily on hardware, and hardware can hardly be improved by AI. AI can optimize software and data analysis, but it cannot make up for fundamental hardware limitations.

*Good chemists with solid intelligence and great experimental skills will never be replaced by AI, and I believe generative AI will be a good friend of them.*

*—Eiichi Nakamura*

## LIFE STORY AND ADVICE FOR THE YOUNG

***NSR*:** Japan has a fine tradition and outstanding achievements in basic scientific research. In what ways has the Japanese education and research system aided your personal career development?

***Nakamura*:** When I was young, Japanese education was somewhat similar to the American system. Students did not focus too much on rigid academic studying at a young age, almost everyone had diverse hobbies until around the age 15, after which we focused more on preparing for the college entrance examinations. I spent a lot of time on hand-making models. I created a full detailed railway model during my high school time. It took me one and a half years, several hundred hours, even while preparing for the college entrance exams. I also loved painting and music.

My high school was one of the best schools in Japan, but the teachers discouraged us to study. They were outstanding teachers; some were also professional painters or musicians, and many of them later became university professors. I did all kinds of things outside of studying with my friends and teachers. We went to the 3000-m-class mountains, held barbecues, organized research seminars, and played music together.

Things have changed a lot nowadays, not only in Japan, but everywhere, even in the US. No one can tell whether these changes are good for scientific research or not. But I think that to foster excellent scientists, no one should force students to work on school subjects. Develop broad interest and do not study just from books. Common sense and practical expertise are also important. Therefore, I fully support China’s recent policies to reduce the academic burden on students.

***NSR*:** What advice would you give to young researchers?

***Nakamura*:** Some people say the new generation in Japan lives a more relaxed life, and this phenomenon is also common in other countries, including China. It is reasonable because after World War II, the regional environment has been stable and parents today are richer than previous generations, so young people enjoy more secure and comfortable lives.

I was born 6 years after the World War II. That unique historical environment nurtured strong curiosity about what would happen, and everyone had a strong internal motivation to improve themselves and explore the world. Such motivation and curiosity are extremely important for outstanding scholars. But now, young people have far less such motivation and for many of them, learning has become a formality only for scores and numbers.

On the other hand, we are now living in an extremely information-rich era, which makes it easy for the young to continuously compare with others and may suffer from pressure. So, my first advice is: do not compare yourself with others, do not overplan the future, and focus on what you are doing now.

I got married with my wife at the end of my Ph.D., when I was 26. I decided to go to Columbia University in New York City for postdoc, and we went there without knowing what would happen.

I did not choose my university—the exam of the University of Tokyo was cancelled so I went to TIT; I did not choose my Ph.D. mentor—I wanted to work with Prof. Mukaiyama, while he moved to the University of Tokyo and left me behind at TIT as a student of one of his former students. So, 2 years before I finished my Ph.D., I decided to choose my postdoc mentor.

At that time, I actually turned down an assistant professor position offer in Japan. My mentor asked me, ‘Do you know what is going to happen after your postdoc? You may not be able to find a job back in Japan.’ I replied, ‘I might find an industrial or academic job in the US, or I might luckily return.’ He pressed further: ‘What if none of these options work out?’ I told him: ‘I can come back and work as an English teacher.’ He said that was perfectly fine, and that I could do whatever I wanted.

My wife and I never overthought our future back then. We took life as it came. It’s the same for coming here (Nankai). We don’t know what will happen. We simply followed our interests. My wife is very curious and eager to explore the city of Tianjin; she has been learning Chinese for 1 year and enjoys every day of it. Our life now feels similar to our time in New York City 50 years ago. We had no plans for the future, yet every day was happy.

In the past, life was full of turmoil and completely unpredictable. No one could plan ahead, so people simply lived in the moment. Today’s younger generation faces a

*Do not compare yourself with others, do not overplan the future, and focus on what you are doing now.*

*—Eiichi Nakamura*

different world, but maybe it’s still good not to overplan the future or set your goals too high. Just **s**et achievable goals that are slightly higher than your current level. Keep making small progress, and eventually you will become better.

Besides this first advice, I have two more pieces of advice for young researchers.

One is that if you dislike writing grant applications, you may not be eligible for academic research. I spend plenty of time writing papers and grant proposals and I like doing it. I believe this is an essential skill for scientists. Because grant papers are appeals to the government and the public, you need to believe in yourself first, and then convince others that your research is valuable and worth investing in.

The other one is that management is very important, probably more important than scientific research itself. This is a view shared by my senior colleagues, and is especially essential for young scientists. Actually, laboratory management is my biggest challenge right now in Nankai. I am truly grateful for all the support from my Chinese colleagues. They are helping me to navigate China’s academic environment that is not quite familiar to me.

***NSR*:** How important is intuition in your research?

***Nakamura*:** I believe intuition only comes after thorough preparation. It is like the famous saying: ‘luck favors the prepared mind.’

***NSR*:** For scientists, what are the preparations needed?

***Nakamura*:** You need good knowledge and good techniques. The same logic applies to music. I play the flute but I am not a professional musician. When I practice music, I keep thinking and practicing. I try to understand the music, and then make it real. This mirrors scientific research: you must first understand your research topic, and then implement it with solid techniques.

Technique is necessary but not sufficient. Amateur learners often overemphasize technique, but technique alone is never enough. You need a clear vision of what you want to achieve, whether in music or research.

Besides, not all music is suitable for amateurs. Most of the 19th-century romantic music is extremely technically demanding and structurally complex, designed only for professionals. I avoid overly complex works after Schubert, Schubert is already complex enough for me, as they demand professional-level technique and in-depth comprehension.

The same applies to chemistry. A good professional research topic should be both academically important and highly challenging, iron catalysis as an example. These are the fields that enable true zero-to-one innovation, which can be achieved only through steady, step-by-step exploration.

## A CONTINUING STORY WITH CHINA

***NSR*:** When did your collaboration with Chinese scientists begin?

**Figure 3:**
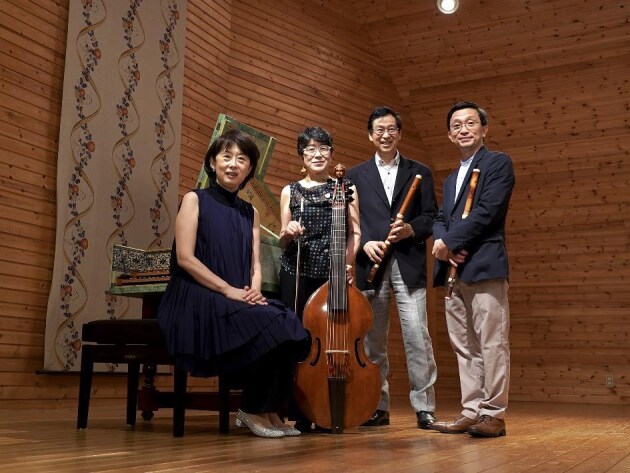
A music ensemble of Prof. Nakamura with his wife and friends. Second from right: Prof. Nakamura with his flute. Third from right: his wife Yoko with viola da gamba in her hand. Photo provided by Prof. Nakamura.

***Nakamura*:** My collaboration with Chinese researchers started in 1993. I met the late Chinese Academy of Sciences (CAS) academician Lixin Dai (戴立信), a distinguished Chinese scholar who passed away in 2024 at the age of 100. Prof. Dai was introduced to me at a meeting by Prof. Ryoji Noyori (野依良治), a Nobel laureate. During my Ph.D., I published a paper on the exact same research topic that Prof. Noyori was studying at the time.

At that time, I was 42 years old, and Prof. Dai was nearly 60. Prof. Noyori recommended me as a rising star in Japanese academia and suggested that Prof. Dai show me around China. So, in May 1994, Prof. Dai invited me to the CAS Shanghai Institute of Organic Chemistry, followed by a visit to the CAS Institute of Chemistry in Beijing. I still remember the scene back then: Beijing was filled with floating catkins, far more than that of today.

Prof. Dai and I met on many occasions after that. I received regular invitations and also invited him to academic conferences in Japan. We met more than 10 times globally over the years.

We built a very close academic and personal friendship, and this friendship inspired me to launch an academic conference for organic chemists across East Asia. I consulted Prof. Dai and Henry Nai Ching Wong (黄乃正), a prestigious Hong Kong chemist who was elected a CAS academician in his early forties and is now in his seventies.

With their support, I founded the annual conference in 2001 (the Tateshina Conference on Organic Chemistry). The conference introduced me to many Chinese scientists. Henry Hong invited five outstanding young scholars around 35 years old each year. In the early 2000s, these young researchers had limited funding, so we covered their travel expenses. I hosted this conference for 12 years and invited roughly 60 young Chinese scholars, nearly half of whom have grown into academicians, such as Kuiling Ding (丁奎岭), Lizhu Wu (吴骊珠), Biao Yu (俞飚), Dawei Ma (马大为), and Shuli You (游书力). Today, they are mostly in their late fifties to mid-sixties, forming a solid and long-term academic connection between China and Japan.

**Figure 4:**
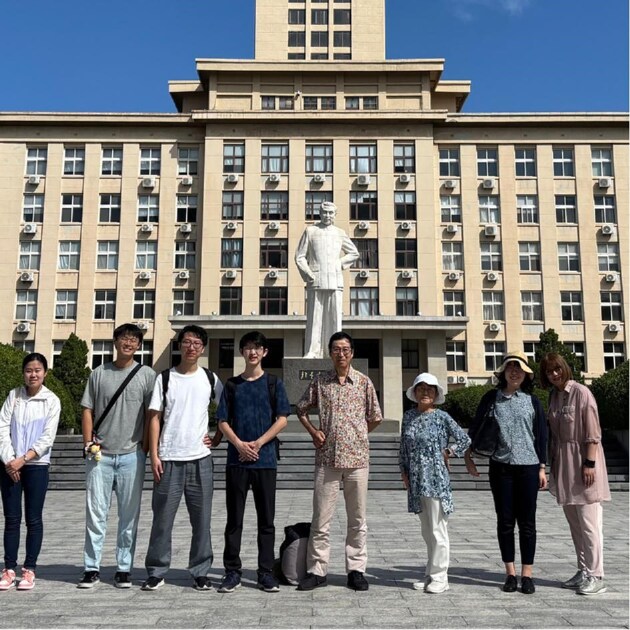
The very initial members of Prof. Nakamura’s new lab at Nankai. Photo provided by Prof. Nakamura.

These conference participants later recommended excellent postdocs to my group. So far, six of them have become recipients of the National Outstanding Youth Science Fund, including Shoufei Zhu (朱守非), who is currently the Vice President of Nankai University and the one who brought me here. I hope my stay at Nankai will further enrich my academic experience and collaborations.

***NSR*:** How did you decide to come to Nankai?

***Nakamura*:** Again, this is ‘beyond expectation’. I was almost going to Sichuan University in Chengdu 3 or 4 years ago, but it didn’t work out. Then, this happened all of a sudden. Prof. Shoufei Zhu became a Vice President of the university and head of the Frontiers Science Center for New Organic Matter, and maybe he was looking for someone to join.

I received an honor from the Emperor in 2024 and we had a symposium at late August 2024. I invited my former alumni, including Zhu. He talked to my team and learned that I could go to China or anywhere if there was a good opportunity. He arranged everything almost immediately, and 3 months later, on mid-December, I met the Chair of the University Council at the main campus. They showed me the new building of the center, and told me to ‘choose whichever lab you wish’, so we chose our new laboratory and that’s how it started.

***NSR*:** I noticed that most of the new lab is still empty. What kind of support had Nankai provided you, and when will the lab be filled?

***Nakamura*:** We need time to recruit students. I plan to develop the lab steadily over the next few years. The school has done its utmost to assist my research. They have tried their best to facilitate the administrative procedures, and overall the progress has been remarkably fast.

We are applying for government financial support to procure an electron microscope as soon as possible. The equipment is very expensive and procuring and installing it will take about 1 year. Before that I can use the microscopes in Tokyo and Nankai, but having a dedicated microscope for our lab would be ideal.

***NSR*:** What are your research plans in Nankai?

***Nakamura*:** I want to do something totally new. Since we are starting a new lab, so once we have a new team of people and a new environment, we can find something new. I want to achieve what Prof. Mukaiyama called ‘quantum leap from 0 to 1’.

## Notes

Weijie Zhao is an NSR news editor based in Beijing. The documentary mentioned at the beginning of the interview: https://www.bilibili.com/video/BV1CGpBeZEMz/?vd_source=7bfa46949e768f818901d74d1db54c22

